# The Activity-Integrated Method for Quality Assessment of Reduning Injection by On-Line DPPH-CE-DAD

**DOI:** 10.1371/journal.pone.0106254

**Published:** 2014-09-02

**Authors:** Yan-xu Chang, Jiao Liu, Yang Bai, Jin Li, Er-wei Liu, Jun He, Xiu-cheng Jiao, Zhen-zhong Wang, Xiu-Mei Gao, Bo-li Zhang, Wei Xiao

**Affiliations:** 1 Tianjin State Key Laboratory of Modern Chinese Medicine, Tianjin University of Traditional Chinese Medicine, Tianjin, China; 2 State Key Laboratory of New-tech for Chinese Medicine Pharmaceutical Process, Kanion Pharmaceutical Co., Ltd, Lianyungang, China; Columbia University, United States of America

## Abstract

A sensitive on-line DPPH-CE-DAD method was developed and validated for both screening and determining the concentration of seven antioxidants of Reduning injection. The pH and concentrations of buffer solution, SDS, β-CD and organic modifier were studied for the detection of DPPH and seven antioxidants. By on-line mixing DPPH and sample solution, a DPPH-CE method for testing the antioxidant activity of the complex matrix was successfully established and used to screen the antioxidant components of Reduning injection. Then, antioxidant components including caffeic acid, isochlorogenic acid A, isochlorogenic acid B, isochlorogenic acid C, chlorogenic acid, neochlorogenic acid and cryptochlorogenic acid were quantified by the newly established CE–DAD method. Finally, the total antioxidant activity and the multiple active components were selected as markers to evaluate the quality of Reduning injection. The results demonstrated that the on-line DPPH-CE-DAD method was reagent-saving, rapid and feasible for on-line simultaneous determination of total pharmacological activity and contents of multi-components samples. It was also a powerful method for evaluating the quality control and mechanism of action of TCM injection.

## Introduction

Traditional Chinese medicines (TCMs) have shown an increasing prospect in recent years as an alternative therapy. Considering that TCMs usually contain one or more substances, multi-ingredient methods for assessing the quality of TCMs are important in order to meet the clinical requirements of safety and efficacy. Some common methods of quality control of TCMs include High-Performance Liquid Chromatography (HPLC) [1]–[3], Ultra-High Performance Liquid Chromatography (UPLC) [4], Gas Chromatography-Mass Spectrometry (GC-MS) [5], HPLC-MS [6] and UPLC-MS [7]. These methods are limited in the determination of the chemical contents of TCMs and they fail to reflect the comprehensive pharmacological effect. Taking these problems into account, it has become imperative to develop a new method of linking pharmacological effects and quality control closely. In light of these, the dual-standard quality assessment, which was defined as a method to evaluate the quality using the total activity and contents of multi-ingredients, was proposed to evaluate the quality of TCMs.

Reduning injection made from extracts of *Gardenia jasminoides Ellis*, *Artemisia annua* L. and *Lonicera japonica* Thunb. is a widely used TCM preparation for the treatment of common cold, cough, acute upper respiratory infection and acute bronchitis in the clinic [8]. The prospective clinical trial demonstrated that Reduning injection could be safely used in the treatment of severe hand, foot, and mouth disease (HFMD) [9]. Clinical studies reported that Reduning injection was safe and effective in curing pneumonia [8]. Reactive oxygen species (ROS) are believed to be crucial in the induction of lung damage caused by pneumonia, while therapeutic agents that could effectively scavenge ROS may prevent or reduce the deleterious effects of pneumonia. Reduning injection could increase the activity of superoxide dismutase (SOD) in lungs of rats treated with lipopolysaccharide [8]. Thus, Reduning injection has antioxidant properties. From available literatures, the components which possess antioxidant properties in Reduning injection have not yet been reported. Therefore, it is considered paramount to investigate its antioxidant properties.

Free radical species such as ABTS (2, 2-azino-bis (3-ethylbenzthiazoline-6-sulfonic acid) and DPPH (1, 1-diphenyl-2-picrylhydrazyl) were primarily selected to test antioxidant capabilities of ingredients or extracts of TCMs by spectrophotometry [10]. Recently, HPLC-DPPH [11] and TLC-DPPH [12] methods were reported to analyze the antioxidant activity of ingredients in extracts of TCMs. Although these analytical methods prevented interference from color pigments of analytes, they often involved usage of large amount of organic reagents which were considered harmful to environment. As a powerful analytical technique, capillary electrophoresis (CE) has gained much attention in separation science [13], since it owned the advantages such as simple preparation process, short analysis time, excellent efficiency and few organic reagents. Capillary electrophoresis with the Diode Array Detector (CE-DAD) method has been used to separate components and successfully applied to quality control of herbal medicines [14]–[16]. In this study, an on-line DPPH-CE-DAD method was developed and validated for the determination of the antioxidant activity of Reduning injection and its quality control.

To our knowledge, the on-line DPPH-CE-DAD method for the analysis of Reduning injection has not been reported yet in literature. In this study, CE was used to separate the chemical components of Reduning injection and then further used to develop the antioxidant activity-integrated method of this herbal preparation. The antioxidant activity-integrated method was used to evaluate the quality of different batches of the sample. The feasibility and precision of this on-line DPPH-CE-DAD method for quality control has been discussed in our report. The on-line DPPH-CE-DAD could not only determine total antioxidant activity of samples, but also screen active components rapidly from the complex Reduning injection. Furthermore CE-DAD could be used to analyze the contents of multiple active components in Reduning injection. This antioxidant activity-integrated method obtained by on-line DPPH-CE-DAD will become an advantageous tool for quality control of herbal medicines.

## Materials and Methods

### Chemicals and Reagents

Seven reference compounds including caffeic acid, isochlorogenic acid A, isochlorogenic acid B, isochlorogenic acid C, chlorogenic acid, neochlorogenic acid and cryptochlorogenic acid were purchased from Chengdu Must Bio. Sci. and Tec. Co. Ltd. (Chengdu, China). 25 batches of Reduning injections were offered from Jiangsu Kanion Pharmaceutical Co. Ltd. (Lianyungang, China). Deionized water used for sample preparations and buffer solutions was provided by a Milli-Q Academic ultra-pure water system (Millipore, Milford, MA, USA). Acetonitrile (ACN) and methanol were purchased from Merck (Germany). DPPH (2,2-Diphenyl-1-picrylhydrazyl) was purchased from Sigma (USA). All other chemicals were of reagent grade.

### Apparatus and conditions

All experiments were performed on an Agilent CE system equipped with a Diode Array Detector (Waldbronn, Germany). Instrumental control and data analysis were operated by Agilent ChemStation software. Separations were performed in a 60.5 cm total length (52 cm to the detector) and 50 µm i.d. bare fused-silica capillary (Ruifeng, Heibei, China). New capillaries were flushed sequentially with 1.0 M NaOH, 0.1 M NaOH and deionized water (10 min each). Prior to every separation, the capillary was conditioned by rinsing with 0.1 M NaOH and deionized water followed by the background electrolyte (BGE) (3 min each). After the last run of each day, capillaries were washed orderly with 0.1 M NaOH (10 min) and deionized water (5 min). BGEs in vials were replenished for every injection to obtain the highest reproducibility of the migration times.

### Preparation of standard solutions and samples

All standard phenolic acids and DPPH were individually dissolved with methanol. Fresh DPPH solution was prepared on each day of analysis at a concentration of 500 µg/mL and stored in the dark. The mixed standards were dissolved in 50% methanol at a stock concentration of 1 mg/mL. A 200 µL aliquot of Reduning injection was diluted independently to 10 mL with the diluent (methanol: water 50∶50, v/v). Standard solutions were stored at 4°C. All solutions were filtered prior to use through a 0.22 µm nylon syringe filter.

### Preparation of quality control samples

Quality control (QC) samples of caffeic acid, isochlorogenic acid A, isochlorogenic acid B, isochlorogenic acid C, chlorogenic acid, neochlorogenic acid and cryptochlorogenic acid were prepared at low, medium and high concentration levels by dissolving appropriate mixed standard solutions in 50% methanol, respectively.

### Condition of on-line DPPH-CE-DAD method

The total antioxidant activities were tested by on-line DPPH-CE-DAD method. Sample solution was injected hydrodynamically into a bare capillary tube at 50 mbar pressure for 5 s and pure DPPH (500 µg/mL) solution was injected immediately after the sample injection in the same way as the sample. Subsequently, a 25 kV voltage was applied with positive polarity setting and the temperature of capillary was maintained at 22°C. The buffer solution for detecting the peak of DPPH to evaluate total antioxidant activity was 20 mM NaH_2_PO_4_ (pH 6.0) – 50 mM sodium dodecyl sulfate (SDS). The pH of BGEs for solution of DPPH was adjusted by 1.0 M NaOH. The wavelength of detection for DPPH was 517 nm, respectively.

### Condition of CE-DAD method for determining multi-components

CE-DAD was used to separate and determine the multiple active components in Reduning injection. Sample solution was injected hydrodynamically at 50 mbar pressure for 5 s. A 25 kV voltage was applied with positive polarity setting and the temperature of capillary was maintained at 22°C. The aqueous BGE for sample separation and determination consisted of 20 mM NaH_2_PO_4_ (pH 4.2), 10 mM β-cyclodextrin (β-CD) and 5% ACN. The pH of BGEs for sample separation was adjusted by using 1% phosphate. The wavelength of detection for the sample separation was 325 nm.

### Condition of DPPH-CE-DAD method for screening antioxidants of Reduning injection

The injection process is the same as the above-referred DPPH-CE-DAD. The chemical reaction of sample and DPPH was performed in the bare fused-silica capillary before separation of analytes and DPPH was carried out by capillary electrophoresis. DPPH is a radical-containing compound which is neutralized by antioxidant molecules. This reaction changes the absorbance properties of DPPH. Consequently, the magnitude of decrease of the DPPH peak seen in the electrophoretogram can be used to quantify the antioxidant activity of the TCM sample. The buffer solution for screening antioxidants of Reduning injection was 20 mM NaH_2_PO_4_ (pH 4.2), 10 mM β-cyclodextrin (β-CD) and 5% ACN. The wavelength of detection for antioxidants was 325 nm.

## Results and Discussion

### Optimization of on-line DPPH-CE-DAD

DPPH is a well-known radical which is used to determine the antioxidant activity. For the purpose of on-line evaluation of antioxidant activity of Reduning injection, there was a need to detect the DPPH peak in a DPPH solution and an on-line spiked Reduning injection mixture. Some parameters including the pH of buffer, the concentrations of buffer and SDS were optimized to develop a capillary electrophoresis method for detecting DPPH. Each optimized experiment was repeated three times.

The concentrations of phosphate buffer and SDS were maintained at 20 and 50 mM, the different pH values (5.5, 6.0, 6.5, 7.0, 7.5 and 8.0) were evaluated. The results showed that shorter migration time, no tailing, less peak width and more peak area were observed at pH 6.0 (**Figure S1A and Figure S2A**). Therefore, pH 6.0 was selected as the optimized pH of BGE.

The different concentrations of phosphate buffer (10, 20, 30, 40 and 50 mM) were tested for the research under constant instrumentation conditions (pH 6.0, 50 mM SDS, 25 kV, 22°C). The results showed that the electrophoretograms were similar at the different concentrations (20, 30, 40 and 50 mM). When the concentration of phosphate buffer was 10 mM, none of the compounds were detected. Meanwhile, high currents which lead to broadening of the peak because of excessive joule heating were observed at the different concentrations (30, 40 and 50 mM) (**Figure S1B and Figure S2B**). Thus, the optimum concentration of phosphate buffer was set at 20 mM.

Finally, the effect of different SDS concentrations (0, 10, 30, 50, 80, 100 mM) on the performance of DPPH separation was studied. At lower concentration (0 mM), the peak of DPPH was not detected. At higher concentrations (100 mM), the migration time became longer (**Figure S1C**). The least peak width was found when a suitable SDS concentration of 50 mM was used (**Figure S2C**). Thus, 50 mM SDS was chosen as the optimum in the following experiments. Therefore, 20 mM phosphate buffer (pH 6.0)–50 mM SDS were optimized to analyze DPPH which was incubated with the TCM in order to evaluate the antioxidant activities of TCM.

### On-line determination of total antioxidant activity of Reduning injection

**Figure 1:**
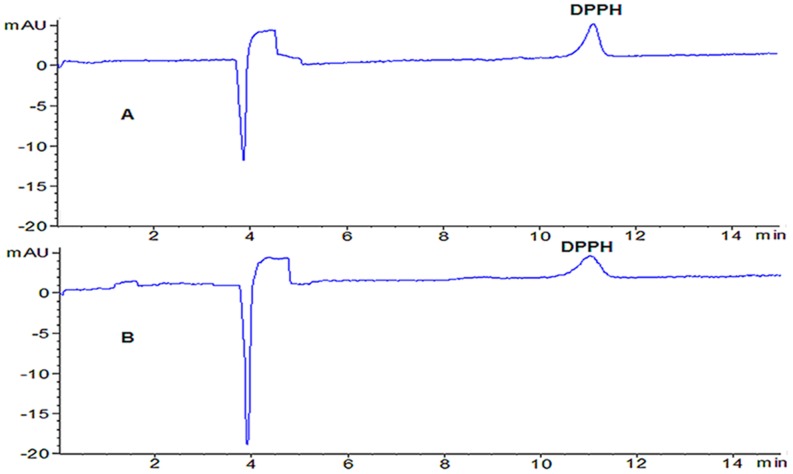
Capillary electropherograms of DPPH: (A) DPPH solution and (B) on-line mixed with Reduning injection (n = 3). Experimental conditions: 50 µm i.d. ×375 µm o.d. ×60.5 cm length (52 cm effective length), uncoated; 20 mM NaH_2_PO_4_ (pH 6.0)–50 mM SDS, voltage, 25 kV; temperature, 22°C; detection wavelength, 517 nm; pressure injection, 50 mbar for 5 s.

The established on-line DPPH-CE-DAD method was used to evaluate total antioxidant activity of Reduning injection. From the **Figure 1**, it can be observed that there was no inference with the peak of DPPH within shorter time (12 min). The peaks of DPPH with no interference in a DPPH solution and an on-line spiked Reduning injection mixture were obtained by DPPH-CE-DAD method. The experiment was repeated three times.

The relative percentage of inhibition of DPPH was calculated using the equation [Inhibition(%) = (P_0_−P_1_)/P_0_×100%] where P_0_ was peak area of DPPH (blank) and P_1_ was peak area of DPPH (on-line spiked Reduning injection). The half maximal inhibitory concentration (IC_50_) of DPPH was selected to assess the antioxidant activity of the different batches of Reduning injection. The IC_50_ values of 25 batches of Reduning injections were listed in **Table 1** (IC_50_ value was represented by the diluted times). The results showed that Reduning injection possessed antioxidant activity. Moreover, the average IC_50_ value of 25 batches was 1049 (diluted times) and relative standard deviation (RSD) was less than 9%, which illustrated that the total antioxidant activity varied a little between each batch considering IC_50_ values. It was concluded that on-line DPPH-CE-DAD could be a powerful method for evaluating total antioxidant activity of complex TCMs.

**Table 1:** Contents of seven compounds in different samples and the result of total IC_50_ values (mg/mL) (n = 3).

Sample	caffeic acid	isochlorogenic acid A	isochlorogenic acid C	chlorogenic acid	isochlorogenic acid B	neochlorogenic acid	cryptochlorogenic acid	IC_50_
091001	0.058	0.314	0.486	7.163	0.564	3.104	3.850	922.5
091002	0.057	0.313	0.483	7.143	0.557	3.114	3.785	890.5
091108	0.054	0.316	0.481	7.106	0.569	3.098	3.832	898.1
091103	0.056	0.313	0.484	7.366	0.575	3.086	3.905	1029
091109	0.057	0.312	0.481	7.062	0.575	3.141	3.919	955.1
091117	0.056	0.314	0.491	7.259	0.573	3.106	3.944	1006
081008	0.055	0.312	0.491	7.387	0.577	3.134	3.935	1047
091104	0.055	0.317	0.486	7.177	0.575	3.106	3.932	971.8
100301	0.057	0.321	0.490	7.483	0.582	3.195	3.908	1173
090908	0.056	0.313	0.492	7.343	0.576	3.169	3.934	1038
090911	0.056	0.313	0.487	7.396	0.568	3.164	3.955	1026
091012	0.057	0.312	0.481	7.233	0.575	3.146	3.929	1001
100206	0.055	0.313	0.488	7.382	0.565	3.194	3.959	1059
090913	0.057	0.317	0.492	7.394	0.572	3.203	3.962	1097
090912	0.057	0.314	0.488	7.422	0.575	3.215	4.000	1189
090907	0.057	0.317	0.492	7.379	0.567	3.200	3.918	1071
090906	0.057	0.316	0.487	7.408	0.576	3.192	3.958	1171
090909	0.056	0.316	0.484	7.505	0.568	3.194	3.930	1186
091206	0.057	0.317	0.488	7.259	0.575	3.164	3.899	1017
100210	0.055	0.316	0.483	7.363	0.577	3.175	3.946	1048
091115	0.056	0.315	0.487	7.396	0.589	3.222	3.966	1173
100101	0.055	0.313	0.491	7.420	0.575	3.179	3.969	1172
091013	0.054	0.311	0.483	7.363	0.570	3.126	3.899	1034
100123	0.056	0.323	0.491	7.319	0.570	3.123	3.900	1024
091014	0.057	0.311	0.464	7.433	0.573	3.205	3.908	1031

### Comparison of on-line DPPH-CE-DAD, HPLC-DPPH and spectrophotometry Methods

The analytical performance of on-line DPPH-CE-DAD and HPLC-DPPH were compared in terms of chemical reagent consumption. Chandrasekar *et al.* had used a mobile phase composition of methanol and water (80∶20, v/v) to establish an HPLC-DPPH method and analysis time was 10 min. Thus, 8 mL methanol (organic solvent) was needed to determine the antioxidant activity of one sample [11]. However, in the developed DPPH-CE-DAD method, the BGE used during the Reduning injection screening contained 5% methanol and only 2.1 µL acetonitrile was used to analyze the antioxidant activity of one sample. Yamaguchi and his team had observed that the color pigments contained in analyzed samples could contribute to the observed difference in IC_50_ values when UV-vis direct spectrophotometric method was used. They also noted that UV-vis direct spectrophotometric method was nonspecific for DPPH [17]. Compared with UV-vis direct spectrophotometric method, the DPPH-CE-DAD method was based on the reduction in DPPH peak area and thus considered specific for DPPH. The superiority of DPPH-CE-DAD method was more evident from the detection of small changes in the DPPH absorbance reflected by the peak area even in the presence of mixtures of TCM extracts and colorants. Therefore, the newly developed on-line DPPH-CE-DAD method was a reagent-saving, rapid, feasible technique and it was also considered as a green technology with no harmful effects to the environment.

### Optimization of CE-DAD method for determining multi-components

To achieve the optimum separation of all seven compounds, the most important parameters were optimized including the pH of buffer, the applied voltage and cassette temperature, the concentrations of buffer, β-cyclodextrin (β-CD) and organic modifier. Each optimized experiment was repeated three times.

### Effect of pH

**Figure 2:**
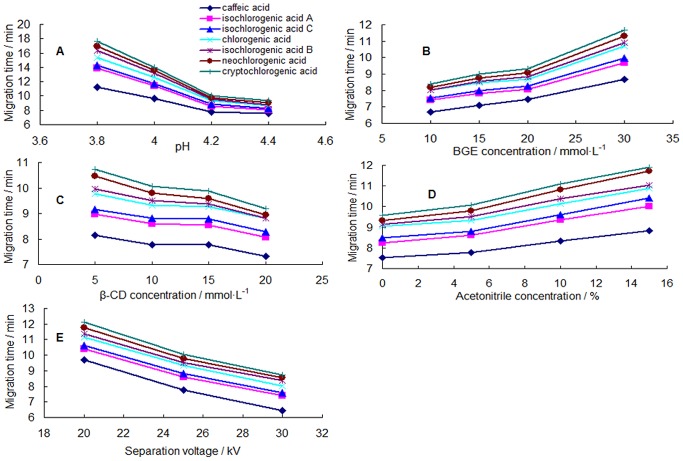
Effects of parameters on the migration time and resolution of seven peaks (n = 3). (A) pH of the phosphate buffer, (B) BGE concentration, (C) β-CD concentration, (D) acetonitrile concentration, (E) separation voltage.

**Figure 3:**
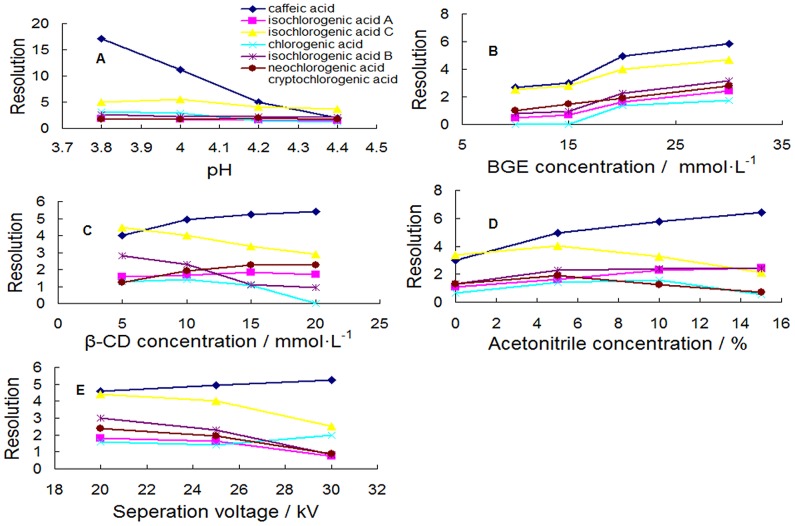
Effects of parameters on the resolution of seven peaks (n = 3). (A) pH of the phosphate buffer, (B) BGE concentration, (C) β-CD concentration, (D) acetonitrile concentration, (E) separation voltage.

The pH which influenced peak efficiency and migration time was of key importance for the separation performance. By regulating the pH of BGE, the migration velocity of weak electrolyte and the velocity of the electroosmotic flow (EOF) changed [17]. The pH of the running buffer ranging from 3.8 to 4.4 were chosen for the study under constant instrumentation conditions (20 mM NaH_2_PO_4_, 10 mM β-CD, 5% ACN, 25 kV, 22°C). **Figure 2A** showed the effect of the pH on the selectivity of the separation and the resolutions (**Figure 3A**). At pH 4.2, appropriate migration was obtained. It was also found that migration time decreased with increasing pH of the buffer in the range of 3.8–4.4. Compared with the electrophoretograms at different pH, the resolution decreased when the buffer pH exceeded 4.2 (**Figure S3A**). Therefore, by comprehensive consideration of the separation and migration time, the optimized pH of BGE was 4.2.

### Effect of BGE concentration

The effect of different concentrations of NaH_2_PO_4_ buffer (10, 15, 20 and 30 mM) on migration and resolution was investigated (**Figure 2B** **and** **Figure 3B**). As can be seen from **Figure 2B**, prolonged migration time was obtained with increase in the concentration of buffer at pH 4.2. There were bad resolutions among all the components in the electrophoretograms of concentration of buffer at 10 and 15 mM (**Figure S3B, S3C**). The improved resolutions were detected in the range of 20–30 mM. By comprehensive consideration of the separation and migration time, 20 mM NaH_2_PO_4_ was selected as optimum concentration.

### Effect of β-CD concentration

β-CD with a relatively hydrophobic cavity which forms inclusion complexes with analytes and an outer hydrophilic layer has been confirmed for the separation of the isomers. The formation of inclusion complexes between the enantiomers and the β-CD is strongly influenced not only by the hydrophobic interaction in the cavity but also by interaction between the hydroxyl groups (or other substituents) on the rim of CDs and substituents of the asymmetric center of the analytes [18]. In our lab, we studied the effect of different β-CD concentrations (5, 10, 15, 20 mM) on the separation performance. The results were shown as **Figure 2C** **and** **Figure 3C**, in spite of the shortened migration time with increasing β-CD concentration, it was necessary to mention a poor resolution at a concentration of 5 mM and a slightly broadened peak shape at 15 and 20 mM from electrophoretograms (**Figure S3D**). When β-CD was treated with 10 mM, all the compounds could achieve a good baseline separation and peak shape.

### Effect of acetonitrile concentration

It is known that organic solvents added into a running buffer can enhance the solubility of the various substance and separation selectivity through changes in the physicochemical properties [18], [19]. The most widely used organic modifier in MEKC is acetonitrile, which is particularly effective in reducing retention factors and expanding the migration window [20]. Hence, acetonitrile ranging from 0% to 15% was chosen to be the organic modifier in this study. There was a poor resolution at a concentration of 0% acetonitrile in the electrophoretogram (**Figure S3E**). Although it caused an increased resolution (**Figure 3D**), prolonged migration times were observed at concentrations more than 5% from **Figure 2D**. Therefore, 5% acetonitrile was chosen as the optimum organic modifier.

### Effect of separation voltage and temperature

The influence of voltages (20, 25, 30 kV) and cassette temperatures (20, 22, 25°C) on peak separation were examined. In despite of the decreasing migration time (**Figure 2E**), all peaks could not reach baseline separation in the electrophoretograms of voltage over 25 kV (**Figure S3F**). The best resolution was observed when a voltage of 25 kV was applied (**Figure 3E**). The suitable migration time and resolutions were similarly observed at 22°C. Therefore, 20 mM phosphate buffer (pH 4.2)–10 mM β-CD - 5% ACN, 25 kV and 22°C were selected to develop the CE-DAD method for determining multi-components of Reduning injection.

### Method validation

#### Linearity, Limits of detection and quantification

The linearity of the method was evaluated by constructing six point calibration curves. Calibration graphs were constructed in the range of 1–100 µg/mL for caffeic acid, isochlorogenic acid A, isochlorogenic acid B and isochlorogenic acid C, 2–200 µg/mL for neochlorogenic acid and cryptochlorogenic acid and 3–300 µg/mL for chlorogenic acid, respectively. Linear regression equations for all standard phenolic acids indicated good correlation with the correlation coefficients (R^2^)>0.9983. The regression equations for caffeic acid, isochlorogenic acid A, isochlorogenic acid B, isochlorogenic acid C, chlorogenic acid, neochlorogenic acid and cryptochlorogenic acid were y = 1.0359x−0.1309, y = 0.7039x−0.9727, y = 0.6993x−0.2756, y = 0.924x−0.9422, y = 0.5662x−6.0336, y = 0.6867x−3.5464, y = 0.6389x−3.3038, respectively (**Table 2**). On the basis of signal-to-noise ratio of 3, limit of detection (LOD) of caffeic acid, isochlorogenic acid A, isochlorogenic acid B and isochlorogenic acid C were 0.25 µg/mL and those of other phenolic acids were 0.50 µg/mL. The limit of quantification (signal-to-noise ratio = 10) is the lowest amount of compound in a sample which can be quantitatively determined. The limits of quantification of caffeic acid, isochlorogenic acid A, isochlorogenic acid B and isochlorogenic acid C were 0.80 µg/mL and all other phenolic acids were 1.50 µg/mL, respectively (**Table 2**).

**Table 2:** The calibration curves, linearity ranges, LODs, LOQs and recoveries of seven compounds (n = 6).

Compounds	Regression equation	R^2^	Linearity range (µg/mL)	LOD (µg/mL)	LOQ (µg/mL)	Recovery	
						Average (%)	RSD (%)
caffeic acid	y = 1.0359x−0.1309	0.9999	1–100	0.25	0.80	104	2.5
isochlorogenic acid A	y = 0.7039x−0.9727	0.9986	1–100	0.25	0.80	101	3.4
isochlorogenic acid C	y = 0.924x−0.9422	0.9990	1–100	0.25	0.80	101	2.2
chlorogenic acid	y = 0.5662x−6.0336	0.9984	3–300	0.50	1.50	99.9	5.0
isochlorogenic acid B	y = 0.6993x−0.2756	0.9985	1–100	0.25	0.80	101	3.4
neochlorogenic acid	y = 0.6867x−3.5464	0.9983	2–200	0.50	1.50	98.1	4.6
cryptochlorogenic acid	y = 0.6389x−3.3038	0.9984	2–200	0.50	1.50	98.3	4.5

#### Precision and accuracy

Precision and accuracy were evaluated by assaying quality control samples containing seven phenolic acids at low, medium, and high concentrations and DPPH solutions (n = 6). The data of intra- and inter-day precision and accuracy were presented in **Table 3**. The intra- and inter-day accuracies of seven phenolic acids were within the range of 91.4%-108%. The RSDs for both intra- and inter-day were below 4.2%. The RSDs for both intra- and inter-day of DPPH were less than 3.0%. The results indicated that the method was accurate, reliable and reproducible.

**Table 3:** Intra-day and Inter-day accuracy and precision, stability of seven compounds and DPPH (n = 6).

Compounds	Concentration (µg/mL)	Intra-day		Inter-day		Stability for 24 h	
		Accuracy (%)	RSD (%)	Accuracy (%)	RSD (%)	Remains (%)	RSD (%)
caffeic acid	2	99.1	4.2	94.6	4.8	94.5	4.8
	10	97.5	3.6	97.9	1.9	96.4	2.1
	50	106	1.2	101	3.7	102	3.2
isochlorogenic acid A	2	103	0.9	102	0.9	102	1.0
	10	96.4	2.2	96.8	0.4	95.1	1.2
	50	98.0	2.1	97.2	0.7	99.7	2.6
isochlorogenic acid C	2	107	2.1	107	0.07	108	1.0
	10	95.1	1.2	95.2	0.6	94.5	1.0
	50	101	1.8	103	1.6	102	1.8
chlorogenic acid	6	96.6	2.1	96.4	0.7	96.3	0.7
	30	94.5	0.2	93.9	0.7	93.7	1.1
	150	101	1.3	99.8	1.3	101	0.3
isochlorogenic acid B	2	106	2.2	107	0.4	108	1.5
	10	95.4	0.3	96.0	0.8	95.0	0.4
	50	100	2.6	100	0.2	101	1.5
neochlorogenic acid	4	96.2	2.4	95.6	2.7	97.6	1.3
	20	95.3	0.2	94.9	0.6	95.4	0.3
	100	102	1.8	101	1.8	102	0.6
cryptochlorogenic acid	4	92.1	2.1	92.2	0.2	91.4	0.6
	20	93.3	0.8	94.4	1.0	93.1	1.5
	100	101	1.1	99.9	1.5	100	1.2
DPPH	500	-	2.8	-	3.0	-	4.4

#### Stability

The stability of seven phenolic acids during the sample storage and processing procedures was evaluated by analyzing quality control samples at low, medium, and high concentrations. The results of stability studies were presented in **Table 3**. After 24 h storage at 4°C, the accuracies of seven phenolic acids were within the range of 92.2%–107% and the RSDs were below 4.8%. The RSD of DPPH was 4.4%. The above results demonstrated that the developed method could be used to determine phenolic acids in the Reduning injection and DPPH.

#### Recovery

The recovery test was performed by spiking equivalent amount of seven investigated compounds into a certain amount Reduning injection sample. The original and resultant samples were analyzed using the method mentioned above. The recovery was calculated by the formula: Recovery (%)  =  (found amount − original amount)/spiked amount ×100%. The recoveries of seven phenolic acids of Reduning injections mixed with appropriate standard solutions for caffeic acid, isochlorogenic acid A, isochlorogenic acid B, isochlorogenic acid C, chlorogenic acid, neochlorogenic acid and cryptochlorogenic acid were assayed, respectively. The mean recoveries of seven phenolic acids determined ranged between 98.1–104% and the RSDs were below 5.0% (**Table 2**).

### On-line screening of antioxidant of Reduning injection

**Figure 4:**
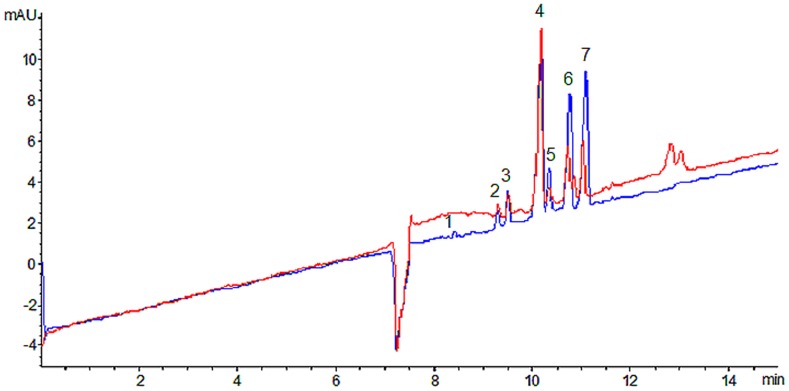
Capillary electropherograms of Reduning injection: Reduning injection (blue) and on-line mixed with DPPH (red) (n = 3). Peaks: 1 =  caffeic acid, 2 =  isochlorogenic acid A, 3 =  isochlorogenic acid C, 4 =  chlorogenic acid, 5 =  isochlorogenic acid B, 6 =  neochlorogenic acid, 7 =  cryptochlorogenic acid. Experimental conditions: 50 µm i.d. ×375 µm o.d. ×60.5 cm length (52 cm effective length), uncoated; 20 mM NaH_2_PO_4_ (pH 4.2) - 10 mM β-CD - 5% (v/v) acetonitrile, voltage, 25 kV; temperature, 22°C; detection wavelength, 325 nm; pressure injection, 50 mbar for 5 s.

**Figure 5:**
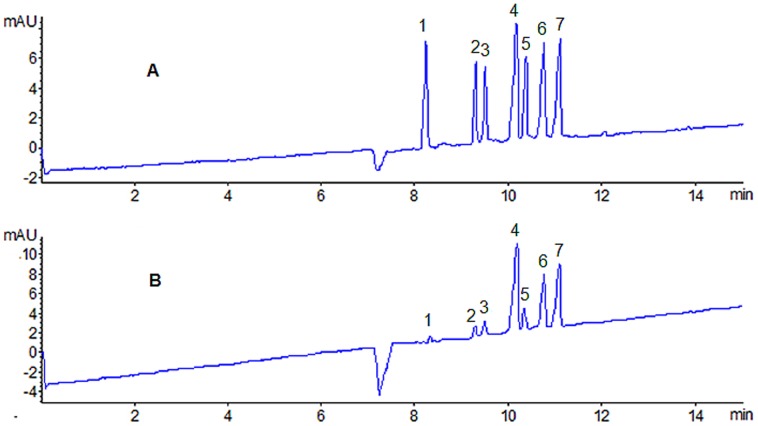
Capillary electropherograms of standard mixture of seven compounds (A) and Reduning injection (B) (n = 3). Peaks: 1 =  caffeic acid, 2 =  isochlorogenic acid A, 3 =  isochlorogenic acid C, 4 =  chlorogenic acid, 5 =  isochlorogenic acid B, 6 =  neochlorogenic acid, 7 =  cryptochlorogenic acid. Experimental conditions: 50 µm i.d. ×375 µm o.d. ×60.5 cm length (52 cm effective length), uncoated; 20 mM NaH_2_PO_4_ (pH 4.2) - 10 mM β-CD - 5% (v/v) acetonitrile, voltage, 25 kV; temperature, 22°C; detection wavelength, 325 nm; pressure injection, 50 mbar for 5 s.

To identify the active ingredients, the various diluted sample solutions and 500 µg/mL DPPH were injected into the on-line CE-DAD system at 50 mbar pressure for 5 s. The experiment was repeated three times. Compared with the electrophoretogram of the sample without DPPH, seven antioxidants were screened based on the decreased peak area (**Figure 4**). These components were identified by comparing with reference standards, including caffeic acid, isochlorogenic acid A, isochlorogenic acid B, isochlorogenic acid C, chlorogenic acid, neochlorogenic acid and cryptochlorogenic acid (**Figure 5**). Thus, these ingredients were the main antioxidant in Reduning injection. It was clearly proved that the antioxidant activity of Reduning injection was the consequence of the effect of these screened compounds. The results observed were consistent with previous studies [21]–[23]. These seven active components were necessarily selected as markers for quality assessment of Reduning injection.

### Method application

The developed method was applied for the determination of seven phenolic acids in 25 batches of Reduning injection and the results were presented in **Table 1**. The typical chromatographic profile of Reduning injection obtained under the above-mentioned conditions was shown in **Figure 5B**. The ranges of quantitative determination for caffeic acid, isochlorogenic acid A, isochlorogenic acid B, isochlorogenic acid C, chlorogenic acid, neochlorogenic acid and cryptochlorogenic acid were 0.054–0.058 mg/ml, 0.311–0.323 mg/ml, 0.464–0.492 mg/ml, 7.062–7.505 mg/ml, 3.785–4.000 mg/ml, 0.557–0.589 mg/ml and 3.086–3.222 mg/ml, respectively. The average of total content of 25 batches was 15.836 mg/ml and the RSD was less than 3.0%. It showed that the seven compounds in 25 batches of Reduning injections varied slightly.

**Figure 6:**
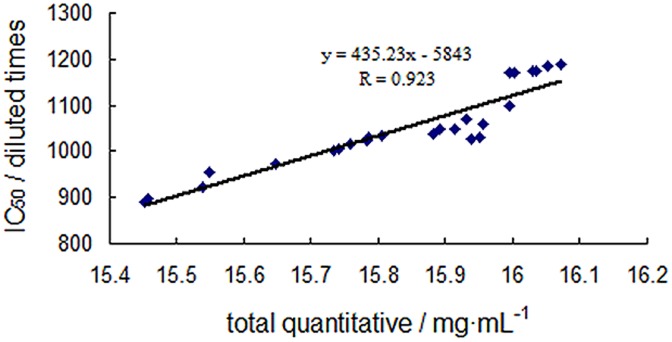
Relationship of the total quantitative and antioxidant activity of Reduning injection.

It is generally known that traditional quality control methods for herbal samples only present the chemical information and could not reflect the real and comprehensive pharmacological information of active constituents, which made these traditional methods less reliable. Therefore, a more reliable approach to evaluate the quality of samples could be the determination of both contents of multiple active components and their activity in contrast to these traditional methods. In our research, we attempted to investigate the relationship between total content and antioxidant activity to evaluate the quality of Reduning injection. As shown in **Figure 6**, there was a good correlation (R = 0.923) between total content of seven compounds and total antioxidant activity across 25 batches of Reduning injection. Therefore, it was important and suitable to choose seven active antioxidant compounds including caffeic acid, isochlorogenic acid A, isochlorogenic acid B, isochlorogenic acid C, chlorogenic acid, neochlorogenic acid and cryptochlorogenic acid as key quality markers to maintain batch-to-batch uniformity and efficacy of Reduning injection. Hence, the combination of total antioxidant activity with multiple active components for dual-standard quality assessment of Reduning injection was concluded to be reliable.

## Conclusions

The dual-standard quality assessment has been proposed to evaluate the quality of Reduning injections with the total antioxidant activity and active multi-ingredients quantification. The antioxidant activity-integrated CE-DAD method was developed for evaluating the total antioxidant activity and screening antioxidant compounds in Reduning injection. By mixing DPPH and sample solution, the on-line DPPH-CE-DAD method was successfully applied to assessing total antioxidant activity and screening the active antioxidant compounds in the samples. Seven components (caffeic acid, isochlorogenic acid A, isochlorogenic acid B, isochlorogenic acid C, chlorogenic acid, neochlorogenic acid and cryptochlorogenic acid) of Reduning injection were characterized as antioxidants and markers and simultaneously determined by CE-DAD method. The results demonstrated that it was reliable to combine total antioxidant activity with multiple active components contents to evaluate the quality of Reduning injection. The on-line DPPH-CE-DAD method was highly efficient due to use of few organic reagents and anti-interference of color pigments in comparison with other existing techniques such as UV-vis direct spectrophotometry, HPLC-DPPH and TLC-DPPH. Hence, it was considered useful in the study of antioxidant activities of other TCMs and valuable for reporting the antioxidant activity of unknown molecules in complex matrix. In contrast to the traditional method of quality control, the present improved method was more available and precise for linking closely total pharmacological activity and contents of multi-components to evaluate quality control of TCM injection.

## Supporting Information

Figure S1 **Effects of parameters on the migration time of DPPH**: (A) pH of the phosphate buffer, (B) BGE concentration, (C) SDS concentration.
(TIF)

Figure S2 **Effects of parameters on the peak width of DPPH**: (A) pH of the phosphate buffer, (B) BGE concentration, (C) SDS concentration.
(TIF)

Figure S3 **Some electrophoretograms of standard mixture of seven compounds with poor resolution in different experimental conditions**: (A) pH = 4.4, 20 mM NaH_2_PO_4_, 10 mM β-CD, 5% (v/v) acetonitrile, 25 kV, (B) pH = 4.2, 10 mM NaH_2_PO_4_, 10 mM β-CD, 5% (v/v) acetonitrile, 25 kV, (C) pH = 4.2, 15 mM NaH_2_PO_4_, 10 mM β-CD, 5% (v/v) acetonitrile, 25 kV, (D) pH = 4.2, 10 mM NaH_2_PO_4_, 5 mM β-CD, 5% (v/v) acetonitrile, 25 kV, (E) pH = 4.2, 10 mM NaH_2_PO_4_, 10 mM β-CD, 0% (v/v) acetonitrile, 25 kV, (F) pH = 4.2, 10 mM NaH_2_PO_4_, 10 mM β-CD, 5% (v/v) acetonitrile, 30 kV.
(TIF)

## References

[1] Zhao L, An Q, Qin F, Xiong Z (2014) Simultaneous determination of six constituents in the fruit of Acanthopanax sessiliflorus (Rupr. et Maxim.) Seem. by HPLC-UV. Nat Prod. Res. 28: 500–502. 24438013 10.1080/14786419.2013.877904

[2] Jian ZY, Wang WQ, Xu GF, Meng L, Hou JL (2013) Comprehensive quality evaluation of Chishao by HPLC. Nutr Hosp 28: 1681–1687. 24160233 10.3305/nh.2013.28.5.6587

[3] Tian S, Yu Q, Wang D, Upur H (2012) Development of a rapid resolution liquid chromatography-diode array detector method for the determination of three compounds in Ziziphora clinopodioides Lam from different origins of Xinjiang. Pharmacogn Mag 8: 280–284. 24082631 10.4103/0973-1296.103653 PMC3785165

[4] Naldi M, Fiori J, Gotti R, Périat A, Veuthey JL, et al (2014) UHPLC determination of catechins for the quality control of green tea. J Pharm Biomed Anal 88: 307–314. 24103292 10.1016/j.jpba.2013.08.054

[5] Hu YC, Kong WJ, Yang XH, Xie LW, Wen J, et al.. (2013) GC-MS combined with chemometric techniques for the quality control and original discrimination of Curcumae longae rhizome: Analysis of essential oils. J Sep Sci. DOI: 10.1002/jssc.201301102. 10.1002/jssc.201301102 24311554

[6] Liang J, Wu WY, Sun GX, Wang DD, Hou JJ, et al (2013) A dynamic multiple reaction monitoring method for the multiple components quantification of complex traditional Chinese medicine preparations: Niuhuang Shangqing pill as an example. J Chromatogr A 1294: 58–69. 23647610 10.1016/j.chroma.2013.04.016

[7] Li TX, Hu L, Zhang MM, Sun J, Qiu Y, et al (2014) A sensitive UPLC-MS/MS method for simultaneous determination of eleven bioactive components of Tong-Xie-Yao-Fang decoction in rat biological matrices. J Chromatogr B Analyt Technol Biomed Life Sci 944: 90–100. 10.1016/j.jchromb.2013.11.015 24295908

[8] Tang LP, Xiao W, Li YF, Li HB, Wang ZZ, et al (2014) Anti-inflammatory effects of Reduning injection on lipopolysaccharide-induced acute lung injury of rats. Chin J Integr Med 20: 1–9. 10.1007/s11655-014-1758-x PMC7101712 24916807

[9] Li X, Zhang X, Ding J, Xu Y, Wei D, et al.. (2014) Comparison between Chinese herbal medicines and conventional therapy in the treatment of severe hand, foot, and mouth disease: a randomized controlled trial. Evid Based Complement Alternat Med. DOI: 10.1155/2014/140764. 10.1155/2014/140764 PMC3955630 24719639

[10] de Oliveira RG Jr, Souza GR, Guimarães AL, de Oliveira AP, Silva Morais AC, et al (2013) Dried extracts of Encholirium spectabile (Bromeliaceae) present antioxidant and photoprotective activities in vitro. J Young Pharm 5: 102–105. 24396251 10.1016/j.jyp.2013.08.005 PMC3812888

[11] Chandrasekar D, Madhusudhana K, Ramakrishna S, Diwan PV (2006) Determination of DPPH free radical scavenging activity by reversed-phase HPLC: a sensitive screening method for polyherbal formulations. J Pharm Biomed Anal 40: 460–464. 16297590 10.1016/j.jpba.2005.07.042

[12] Cieśla Ł, Kryszeń J, Stochmal A, Oleszek W, Waksmundzka-Hajnos M (2012) Approach to develop a standardized TLC-DPPH• test for assessing free radical scavenging properties of selected phenolic compounds. J Pharm Biomed Anal 70: 126–135. 22749343 10.1016/j.jpba.2012.06.007

[13] Cao J, Qu HB, Cheng YY (2010) The use of novel ionic liquid-in-water microemulsion without the addition of organic solvents in a capillary electrophoretic system. Electrophoresis 31: 3492–3498. 20922759 10.1002/elps.201000168

[14] Cao J, Li P, Yi L (2011) Ionic liquids coated multi-walled carbon nanotubes as a novel pseudostationary phase in electrokineticchromatography. J Chromatogr A 1218: 9428–9434. 22119676 10.1016/j.chroma.2011.11.013

[15] Cao J, Li P, Chen J, Tan T, Dai HB (2013) Enhanced separation of Compound Xueshuantong capsule using functionalized carbon nanotubes with cationic surfactant solutions in MEEKC. Electrophoresis 34: 324–330. 23161282 10.1002/elps.201200101

[16] Rodríguez J, Castañeda G, Contento AM, Muñoz L (2012) Direct and fast determination of paclitaxel, morphine and codeine in urine by micellar electrokinetic chromatography. J Chromatogr A 1231: 66–72. 22365566 10.1016/j.chroma.2012.02.003

[17] Yamaguchi T, Takamura H, Matoba T, Terao J (1998) HPLC method for evaluation of the free radical-scavenging activity of foods by using 1,1-diphenyl-2-picrylhydrazyl. Biosci Biotechnol Biochem 62: 1201–1204. 9692204 10.1271/bbb.62.1201

[18] Orlandini S, Gotti R, Furlanetto S (2014) Multivariate optimization of capillary electrophoresis methods: A critical review. J Pharm Biomed Anal 87: 290–307. 23669025 10.1016/j.jpba.2013.04.014

[19] Huie CW (2003) Effects of organic solvents on sample pretreatment and separation performances in capillary electrophoresis. Electrophoresis 24: 1508–1529. 12761781 10.1002/elps.200305396

[20] Gottardo R, Bertaso A, Pascali J, Sorio D, Musile G, et al (2012) Micellar electrokinetic chromatography: a new simple tool for the analysis of synthetic cannabinoids in herbal blends and for the rapid estimation of their log P values. J Chromatogr A 1267: 198–205. 23022243 10.1016/j.chroma.2012.08.085

[21] Gašić U, Kečkeš S, Dabić D, Trifković J, Milojković-Opsenica D, et al (2014) Phenolic profile and antioxidant activity of Serbian polyfloral honeys. Food Chem 145: 599–607. 24128520 10.1016/j.foodchem.2013.08.088

[22] Takeoka GR, Dao LT (2003) Antioxidant constituents of almond Prunus dulcis (Mill.) D.A. Webb] hulls. J Agric Food Chem 51: 496–501. 12517116 10.1021/jf020660i

[23] Iwai K, Kishimoto N, Kakino Y, Mochida K, Fujita T (2004) In vitro antioxidative effects and tyrosinase inhibitory activities of seven hydroxycinnamoyl derivatives in green coffee beans. J Agric Food Chem 52: 4893–4898. 15264931 10.1021/jf040048m

