# Supplementary figures and images for: The Activity-Integrated Method for Quality Assessment of Reduning Injection by On-Line DPPH-CE-DAD
Source: PLoS One. 2014 Sep 2;9(9):e106254. doi: 10.1371/journal.pone.0106254 (PMC4152118; doi:10.1371/journal.pone.0106254)

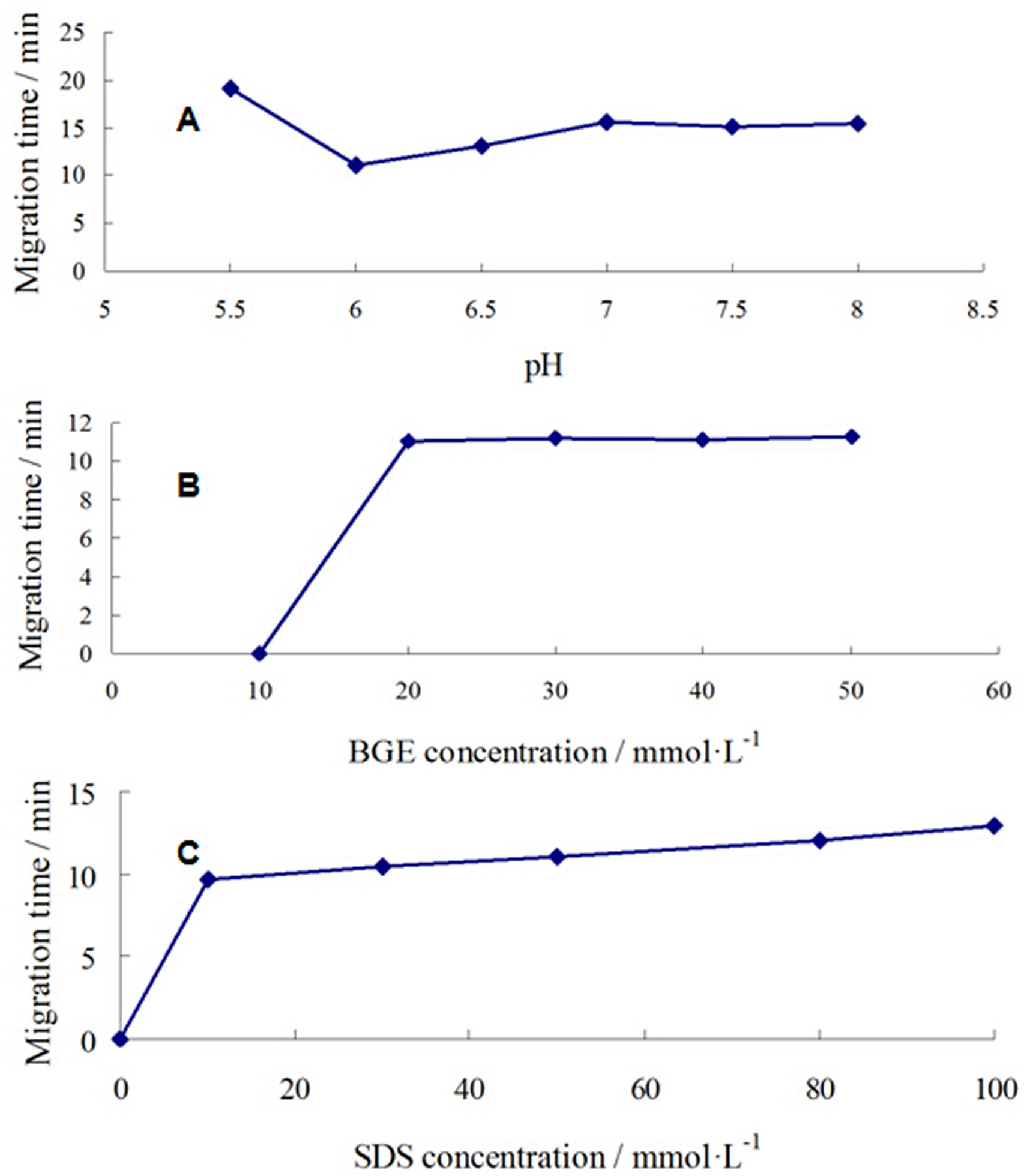

Supplement: Figure S1 — Effects of parameters on the migration time of DPPH: (A) pH of the phosphate buffer, (B) BGE concentration, (C) SDS concentration. (TIF) [file pone.0106254.s001.tif]

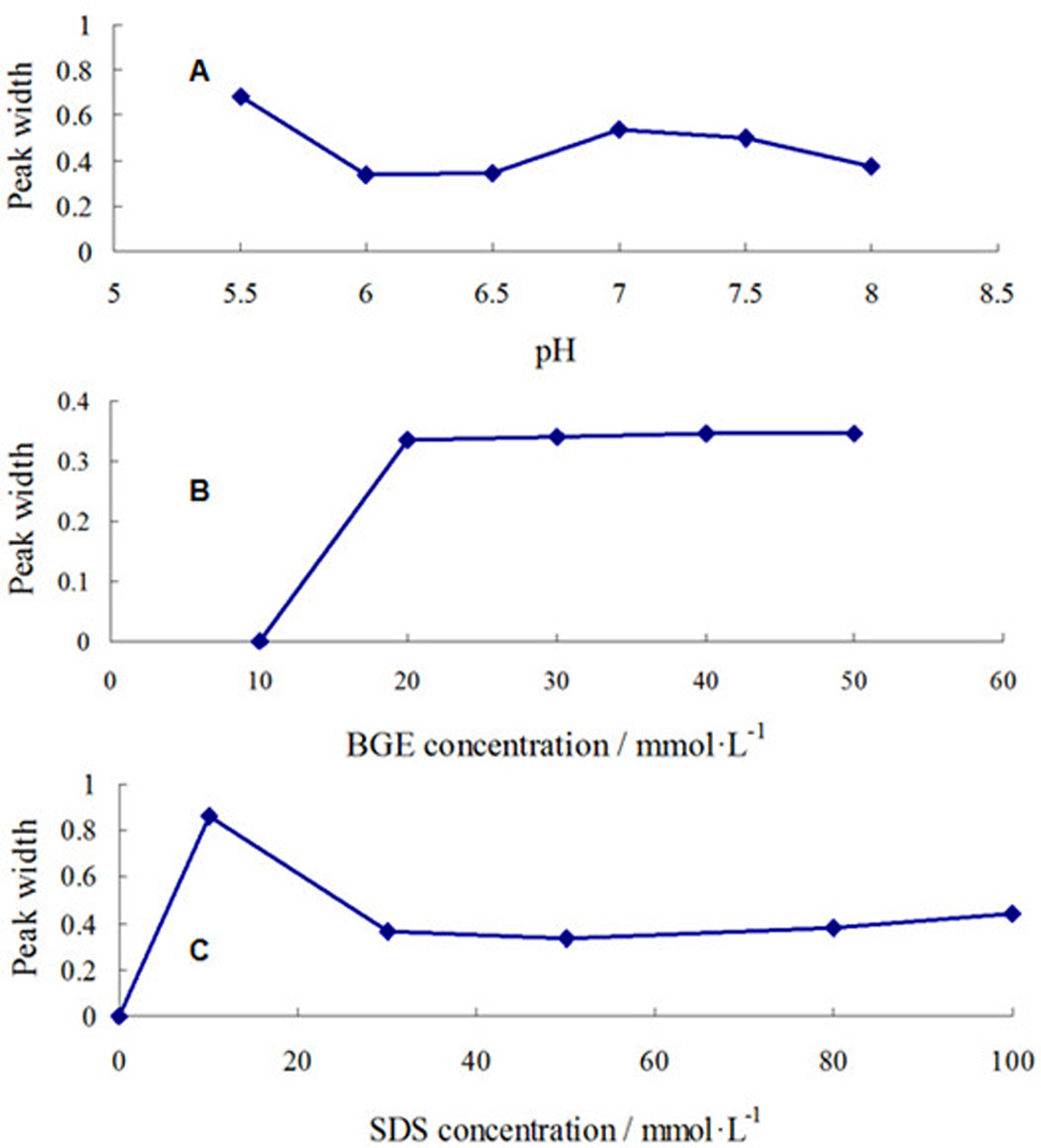

Supplement: Figure S2 — Effects of parameters on the peak width of DPPH: (A) pH of the phosphate buffer, (B) BGE concentration, (C) SDS concentration. (TIF) [file pone.0106254.s002.tif]

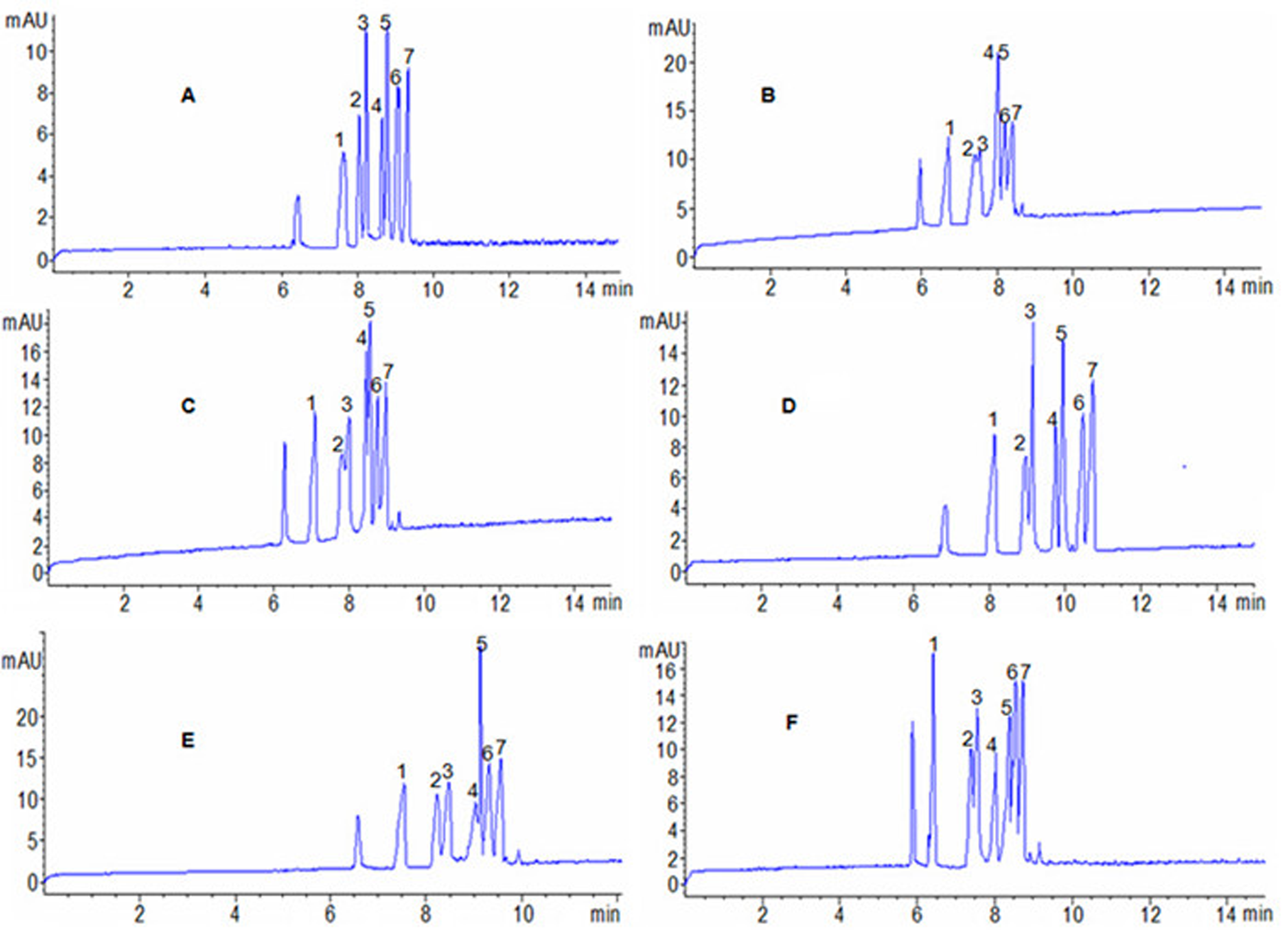

Supplement: Figure S3 — Some electrophoretograms of standard mixture of seven compounds with poor resolution in different experimental conditions: (A) pH = 4.4, 20 mM NaH2PO4, 10 mM β-CD, 5% (v/v) acetonitrile, 25 kV, (B) pH = 4.2, 10 mM NaH2PO4, 10 mM β-CD, 5% (v/v) acetonitrile, 25 kV, (C) pH = 4.2, 15 mM NaH2PO4, 10 mM β-CD, 5% (v/v) acetonitrile, 25 kV, (D) pH = 4.2, 10 mM NaH2PO4, 5 mM β-CD, 5% (v/v) acetonitrile, 25 kV, (E) pH = 4.2, 10 mM NaH2PO4, 10 mM β-CD, 0% (v/v) acetonitrile, 25 kV, (F) pH = 4.2, 10 mM NaH2PO4, 10 mM β-CD, 5% (v/v) acetonitrile, 30 kV. (TIF) [file pone.0106254.s003.tif]
